# Odds of fussy eating are greater among children with obesity and anxiety

**DOI:** 10.1002/osp4.548

**Published:** 2021-08-07

**Authors:** Sigrun Thorsteinsdottir, Anna S. Olafsdottir, Berglind Brynjolfsdottir, Ragnar Bjarnason, Urdur Njardvik

**Affiliations:** ^1^ Department of Pediatrics Landspitali University Hospital Reykjavik Iceland; ^2^ Faculty of Health Promotion, Sport and Leisure Studies University of Iceland Reykjavik Iceland; ^3^ Faculty of Medicine University of Iceland Reykjavik Iceland; ^4^ Faculty of Psychology University of Iceland Reykjavik Iceland

**Keywords:** anxiety, children, fussy eating, obesity, psychopathology

## Abstract

**Background:**

Fussy eating has been associated with autism spectrum disorder (ASD), attention‐deficit/hyperactive disorder (ADHD), anxiety, and depression. Despite these disorders being prevalent in obesity treatment, no studies have been published on the association of fussy eating in children with obesity and these disorders. Understanding fussy eating in children with obesity and comorbid disorders is important as acceptance of healthy foods tends to be low, especially in children with sensory sensitivities.

**Objectives:**

Investigate the prevalence of fussy eating in a cross‐sectional sample of children with obesity and ASD, ADHD, anxiety, and depression; and whether they were more likely to be fussy eaters, comparing those with and without these disorders.

**Methods:**

One hundred and four children referred to family‐based obesity treatment in Iceland 2011–2016, mean age 12.0 (SD = 3.0), mean body mass index standard deviation score 3.5 (SD = 0.9). Binary logistic regression was used to estimate the relationship between fussy eating and disorders, adjusting for medication use.

**Results:**

A large minority (41.6%) were fussy eaters and 48.9% had at least one comorbid disorder. Over a third of children rejected bitter and sour tastes, and 1.9% and 7.9% rejected sweet and salty tastes, respectively. Compared with those without disorders, the odds of being a fussy eater were increased by a factor of 4.11 when having anxiety (95% confidence intervals) (1.02–16.58, *p* = 00.046), adjusting for medication use. The odds of being a fussy eater were not increased for other disorders; ASD, ADHD, or depression.

**Conclusions:**

In children attending obesity treatment, fussy eating was common. Clinical care models in pediatric obesity treatment should address fussy eating, especially in children with anxiety.

## 1 INTRODUCTION

Childhood obesity rates have increased globally in the last decades.^1^ The health consequences of obesity are substantial, especially since children with obesity seem likely to have obesity as adults, with several physical and psychological comorbidities.^2^, ^3^ Childhood overweight and obesity have also been linked to neurodevelopmental disorders (ND) such as autism spectrum disorder (ASD) as well as attention‐deficit/hyperactive disorder (ADHD).^4^, ^5^ Psychological disorders including anxiety and depression are also more commonly seen in children attending obesity treatment and may significantly affect children's quality of life and well‐being.^6^, ^7^ The relationship between obesity and psychological problems such as anxiety and depression seems to be bidirectional, in that psychological difficulties might promote weight gain, and obesity may lead to psychosocial problems.^7^ The combination of having obesity and these disorders may also be associated with uncontrolled or unhealthy eating behaviours.^2^, ^6^, ^7^, ^8^, ^9^

From a public health perspective, concerns about long‐term health consequences of obesity have increased the focus on children's eating behaviors, such as fussy eating.^10^, ^11^ Fussy eating is characterized by a limited variety of foods, that is, a narrow range of preferred foods and rejection of a large proportion of familiar foods (selective/picky eating), and food neophobia, being unwilling to try novel foods, often requiring preparation of separate meals, with strong food likes or dislikes.^10^, ^12^, ^13^, ^14^ Children with fussy eating often reject bitter tastes, which are common in vegetables and medicine.^14^, ^15^, ^16^ The rejection of sour or bitter tastes may be a biological predisposition for most children, although levels of rejection may be raised in children with fussy eating.^17^, ^18^ Furthermore, a study on taste perception in infants showed that accepting sour tastes was associated with higher acceptance of fruits.^19^

Fussy eating is, by some researchers, considered a potential contributing and maintaining factor of obesity in children.^20^ For example, in one study on 4‐6‐year olds, fussy eating weakened the effectiveness of obesity treatment.^21^ Furthermore, a recent study considered treatment of fussy eating a possible mechanism underlying success of family‐based childhood obesity treatment.^22^ The mechanism has been explained in various ways. For example, coercive feeding strategies have been implicated in fussy eaters' overconsumption of food,^23^, ^24^ and parents' feeding of calorically dense and very palatable food, instead of rejected food items like fruit and vegetables which are lower in caloric density.^25^, ^26^ Similarly, children with obesity are more likely to consume larger amounts of calories from sweets and sugary drinks and less likely to consume nutritious foods like fruit and vegetables than children in the healthy weight range.^20^, ^21^, ^27^, ^28^ The association between increased weight and fussy eating has been shown to be relatively stable into adulthood.^29^

Prevalence rates of fussy eating are considered highest among pre‐school children, ranging between 14% and 50%,^30^, ^31^ and lower in later childhood, from 7% to 27%, declining to the lower rates from 6 years of age.^32^ Fussy eating seems less likely, however, to decline in later childhood in children with neurodevelopmental disorders such as ASD and/or ADHD.^33^ In a Swedish study, 40% of 9‐12‐year‐old twins with eating problems were screened positive for ASD and/or ADHD.^34^ One review indicated that 53%–83% of children with ASD, aged 2.5–18.0 years, were reluctant to try new foods, were picky eaters, refused bitter tastes, or had a restricted food repertoire.^14^, ^35^ These continued difficult eating behaviors, especially in children with ASD, may possibly be explained by anxiety, fear, and oppositional behavior.^10^, ^14^, ^36^ Despite the biological predisposition for most children disliking bitter taste,^16^ research on children with ND, especially ASD, has shown sensory sensitivities toward textures and tastes, especially in fibrous foods such as fruit and vegetables.^35^, ^37^, ^38^ This increased sensitivity to sensory experiences, such as taste, smell, and touch, are characteristics that are also highly associated with fussy eating.^39^ Sensory sensitivities may be one of the reasons fussy eaters, particularly those with ASD, often have diets consisting of foods that are bland in colors, and lacking in textures and taste, as well as being low in nutrient density.^34^, ^37^, ^40^ However, although certain stimuli such as textures and taste may be explained by anxiety,^41^ especially in children with ASD,^42^ a recent study indicated many overlapping traits and certain similarities between children with ASD and ADHD in terms of sensory sensitivities in food‐related behaviors.^38^, ^43^ Pressure to eat may also increase anxiety in children, irrespective of disorders.^41^, ^42^, ^44^, ^45^ Furthermore, depression, although not a direct cause of fussy eating, may be associated with decreased, or increased appetite, especially for unhealthy foods, and less acceptance for healthier options such as fruit and vegetables.^46^

Increased appetite may also be due to certain side effects of psychotropic medication.^47^ An increased susceptibility to antipsychotic‐induced, and particularly rapid weight gain, has been widely documented in children with neurodevelopmental and psychological problems.^47^, ^48^, ^49^ The side effects of psychotropic medicine may include associated cardiometabolic abnormalities with central obesity, dyslipidemia, insulin resistance, alteration of metabolism, and systemic inflammation.^4^, ^48^, ^50^ Psychotropic medications have also been associated with increased liver disease severity in pediatric non‐alcoholic fatty liver disease.^49^ Further, the use of antipsychotic medication with methylphenidates in treating complex and severe symptoms of ADHD in children has been increasing with associated weight gain.^51^ However, loss of appetite and reduced weight are also well established in individuals with ADHD using methylphenidates.^52^, ^53^ Despite the known side effects of psychotropic medicine on children's appetites, only one published study exists on the effects of these medicines on altered taste perception of other compounds such as sucrose, resulting in increased or decreased acceptance of certain foods.^54^

As having neurodevelopmental and psychological disorders may be associated with unhealthy eating habits which may in turn lead to overweight or obesity,^5^, ^33^, ^55^, ^56^, ^57^ it is important to investigate aspects of obesity treatment that are less known, such as associations between neurodevelopmental and psychological disorders and fussy eating. To the authors' best knowledge, no previous study has been published on these associations.

The purpose of this study was to investigate fussy eating in a cross‐sectional sample of children attending obesity treatment. The secondary aim was to investigate whether children with obesity and disorders including ASD, ADHD, anxiety, or depression were more likely to be fussy eaters, compared to those who did not have these disorders.

## 2 METHODS

### 2.1 Participants

The original participant population included 297 Icelandic children with obesity who had attended a family‐based multidisciplinary treatment at the Department of Paediatrics, University Hospital in Iceland, between 2011 and 2016. Of the 297 children, parents of 190 provided contact details captured in the hospital's database. Of the 190 families, there were 129 parents who accepted an invitation by the study authors to participate, and a total of 104 parents responded to the questionnaires sent out by the study authors (response rate 54.7%). All participants were Icelandic, and the majority was living in the capital and surrounding regions. To be accepted into the obesity treatment, the children had to be ≥2.5 BMI‐SDS (mean body mass index standard deviation scores). This inclusion criterion was set by the obesity clinic.^58^ No exclusion criteria were set for the present study regarding medication use or diagnosed disorders. Descriptive analyses of the participant population are provided in Table 1.

**TABLE 1:** Characteristics of children in the study (*n* = 104)

	All
	(*n* = 104)
Age, mean (SD), years	12.0 (3.0)
BMI, mean (SD) kg/m^2^	30.4 (5.2)
BMI‐SDS, mean (SD)	3.5 (0.9)
Boys, *n* (%)	54 (51.9)
Medication use^a^	26 (33.3)
Characteristics of fussy eating, *n* (%)	(*n* = 77)
Food neophobia^b^	26 (26.0)
Narrow range^b^	28 (26.9)
Fussy eating^c^	42 (41.6)
Rejects taste^b^	
Bitter	38 (38.5)
Sour	33 (33.3)
Sweet	2 (1.9)
Salty	8 (7.9)
	(*n* = 88)
Disorders^d^, *n* (%)	43 (48.9)
ADHD	28 (31.8)
ASD	11 (12.5)
ASD and ADHD	7 (7.9)
Anxiety	27 (30.7)
Anxiety and ASD	7 (7.9)
Anxiety and ADHD	17 (18.9)
Depression	20 (20.8)
Depression and anxiety	16 (18.2)
Depression and ASD	4 (4.5)
Depression and ADHD	11 (12.5)
Comorbid disorders	
Two disorders	17 (19.3)
Three disorders or more	15 (17.0)
Intellectual disabilities	4 (4.2)
Learning disorders	5 (5.2)
Oppositional defiant disorder	6 (6.3)

*Note:* Frequencies are expressed as mean (SD) and *n* (%).

Abbreviations: ADHD, attention‐deficit/hyperactive disorder; ASD, autism spectrum disorder; BMI, body mass index; BMI–SDS, standard deviation scores; SD, standard deviation.

^a^ *n* = 79.

^b^ Four parents did not provide answers, based on the Kauer et al. questionnaire.^24^

^c^ Combination variable for fussy eating (“Food neophobia” and “Narrow range”).

^d^ *n* = Some children had multiple disorders.

### 2.2 Measures

#### 2.2.1 Background data and child anthropometric measurements

Anthropometric measurements were gained at the children's first visit to the hospital in 2011–2016. The children wore light clothing, were asked to empty all pockets before stepping on the scale, and all were without shoes. Height was measured to the nearest 1.0 mm using a stadiometer (*Ulmer‐Stadiometer*, *Busse Design & Engineering*), and body weight was measured to the nearest 0.1 kg on a digital scale (*M1100 series*, *Marel*). Obesity was defined according to the International Task Force of Obesity.^58^ BMI‐SDS were derived from BMI reference values for Swedish children adjusting to age and sex.^59^

Background information were retrieved from the children's medical files regarding parents' marital status, education level, and occupational status. Information on children's psychological disorders such as anxiety or depression and neurodevelopmental disorders such as ASD or ADHD was also obtained from the medical files by the study authors. Information on whether the children were currently taking any medication was also obtained from the medical files and was coded as “taking medication” or “not taking medication” by the study authors.

### 2.3 Picky eating questionnaire

The Adult Picky Eating Questionnaire by Kauer and colleagues^60^ was used as a basis for a questionnaire designed to obtain a parental report on children's food‐related behavior. The list comprised 30 “True” or “False” statements, such as: “My child does not like to try new foods,” “My child eats from a very narrow range of foods (fewer than 10 different foods),” “My child always rejects sour foods,” “My child always rejects foods that have touched on the plate.” The questionnaire includes the following subscales: *Other eating behaviors; Narrow range; Neophobia; Sensory rejection: taste*; *Sensory rejection: texture; Sensory rejection: appearance; Contact or mixing; Ritualization/repetition; Interest in food/social eating*. For the purpose of this study, one variable from the *Narrow range* subscale was used: “I eat from a very narrow range of foods (fewer than 10 different foods)” and the only item from the *Neophobia* subscale: “I do not like to try new foods.” In addition, *Sensory rejection: taste* was also included in the analyses.

### 2.4 Fussy eating

To represent fussy eating, a combination variable, *Fussy eating*, was used. The variable was made up of *Food neophobia* (“I do not like to try new foods”) and *Narrow range* (“I eat from a very narrow range of foods [fewer than 10 different foods]”).^60^ If parents replied “True” to either or both variables, this was marked as “True” for Fussy eating. Due to a moderate correlation between the two variables (*r* = 0.523, *p* < 0.001), and since they represent the two main forms of fussy eating^10^, ^13^, ^60^, ^61^ and to retain statistical power, this variable was chosen as the dependent variable for the multivariable logistic regression.

### 2.5 Data collection

The data collection for the present study started in January 2016 and was completed in April the same year. Informed written and oral consents were obtained from parents via telephone and email. A questionnaire was then distributed to the parents, and replies were collected via Question Pro (QuestionPro Inc., 2016). Participants were not reimbursed for participating. The research was approved by the Data Protection Authority, The Bioethics Committee in Iceland (VSNb2013010026/03.07).

### 2.6 Statistical analysis

Study data were collected and managed using REDCap (Research Electronic Data Capture) electronic data capture tools hosted at University Hospital, Iceland. All measurements were analyzed with R version 4.0.5 (R Foundation for Statistical Computing). Descriptive statistics were used to characterize the participants (Table 1). Chi‐square and Fisher's exact test were used as appropriate to examine the cross‐tabulation of main characteristics of food‐related behaviors: fussy eating; *Narrow eating*, *Food neophobia*, and the combination variable, *Fussy eating*, based on ASD, ADHD, anxiety, or depression. *Sensory rejection: taste* was inspected by frequencies. Cases with missing data were excluded listwise. Multivariable associations between the combination variable *Fussy eating* and disorders (ASD, ADHD, depression, and anxiety) were assessed using binary logistic regression, adjusting for medication use. Estimated associations were described with odds ratios and 95% confidence interval (CI). No a priori statistical power calculation was conducted for this analysis as sample sizes were based on available data at the time of analyses. Because multiple tests were conducted, alphas were adjusted using Bonferroni correction. After the correction, the remaining significant associations were highlighted (Table 3).

**TABLE 2:** Comparison of parents' estimate of children's food‐related behaviors (“Yes”, “No”) based on disorders

Parents' estimate of children's food‐related behavior																
	Food neophobia^a^						Narrow range^a^					Fussy eating^b^				
Disorders	Yes	No	*χ*2(1)	*φ*	*p*		Yes	No	*χ*2(1)	*φ*	*P*	Yes	No	*χ*2(1)	*φ*	*p*
ASD, *n* = 11	6 (25.0)	4 (6.6)	5.64	0.26	**0.018**		8 (27.6)	3 (5.4)	8.38	0.31	**0.006** ^c^	10 (26.3)	1 (2.0)	11.67	0.36	**0.001** ^c^
Anxiety, *n* = 27	14 (23.0)	12 (50.0)	5.93	0.17	**0.015**		14 (25.0)	11 (37.9)	1.54	0.13	0.215	9 (18.0)	18 (47.4)	8.76	0.31	**0.003** ^c^
ADHD, *n* = 28	17 (25.4)	10 (38.5)	1.56	0.13	0.212		15 (24.6)	13 (40.6)	2.56	0.17	0.109	15 (27.8)	13 (31.0)	0.11	0.03	0.734
Depression, *n* = 20	11 (16.4)	8 (30.8)	2.37	0.16	0.123		10 (16.4)	9 (28.1)	1.78	0.14	0.183	7 (13.0)	13 (31.0)	4.64	0.31	**0.031**

*Note:* Values are expressed as *n* (%). All *p*‐values from *χ* ^2^ test or Fisher's exact test.

Abbreviations: ADHD, attention‐deficit/hyperactive disorder; ASD, autism spectrum disorder.

^a^ Based on Kauer's et al.'s questionnaire.^60^

^b^ Combination variable for fussy eating (“Food neophobia” and “Narrow range”).

^c^ Results significant after correcting for multiple tests.

## 3 RESULTS

As seen in Table 1, the mean age of the 104 participating children was 12.0, SD = 3.5 (range 5.9–18.0 years). Mean BMI‐SDS: 3.5 (SD = 0.9, range 2.5–6.4). Almost half of the participants were girls (48.1%). A third of the parents who had provided information in the children's medical files regarding medication use stated that their children used at least one medication. Of the 88 children who had information on their diagnoses, nearly half of the children (48.9%) had a diagnosed disorder, that is, a neurodevelopmental disorder (ASD, ADHD) with or without psychological disorders (anxiety, depression). Information on children's diagnoses was missing for 16 children. Over a third of the parents had at least an undergraduate degree (38.2%), and 68.8% had a part‐time or full‐time occupation. Nearly a third (30.5%) of the children had divorced parents and split their time between both households (parent background data not shown in Table 1).

### 3.1 Frequencies and characteristics of fussy eating

Just over a quarter of the children were food neophobic, and 26.9% consumed a narrow range of foods. When using the combination variable *Fussy eating*, nearly half (41.6%) of the children were regarded as being fussy eaters. With regards to sensory rejection due to taste, more than a third of the children rejected bitter and sour tastes, and just under 2.0% and 8.0% rejected sweet or salty tastes, respectively (Table 1).

### 3.2 Comparison of parents' estimate of children's food‐related behaviors based on disorders

A chi‐square test for association was conducted between disorders and food‐related behaviors. Fisher's exact test was used where appropriate (Table 2). Significant associations were found between ASD and all measures of fussy eating; *Food neophobia*, *Narrow range*, and the combination variable *Fussy eating*. Significant associations were found for anxiety on *Food Neophobia* and *Fussy eating*. No significant associations were found for ADHD, and only *Fussy eating* was significantly associated with depression. Effect sizes were small in all instances (*φ* 0.03–0.36).

**TABLE 3:** Multivariable associations between having ASD, ADHD, anxiety, or depression versus no disorder, and the combination variable *Fussy eating*, using binary logistic regression analysis (non‐adjusted and adjusted analysis), *n* = 77

	*n*	*β*	Unadjusted OR (95% CI)	*p*	*β*	Adjusted OR (95% CI)	*P*
Medication use							
No	51					
Yes	25				0.95	2.59 (0.69‐9.72)	0.158
Disorders							
None vs	51						
ASD	6	1.75	5.78 (0.49–68.00)	0.163	1.35	3.84 (0.32–46.55)	0.290
None vs							
ADHD	26	−0.76	0.47 (0.12–1.84)	0.277	−0.99	0.37 (0.09–1.56)	0.178
None vs							
Anxiety	22	1.72	5.58 (1.50–20.77)	**0.010**	1.41	4.11 (1.02–16.58)	**0.046**
None vs							
Depression	13	0.49	1.63 (0.31–8.72)	0.560	0.32	1.38 (0.24–7.98)	0.719

Abbreviations: ADHD, attention‐deficit/hyperactive disorder; ASD, autism spectrum disorder; CI, confidence intervals; OR, odds ratio.

### 3.3 Multivariable associations between disorders and fussy eating

A regression analysis was conducted containing the combination variable *Fussy eating* as the dependent variable and disorders (ASD, ADHD, anxiety, and depression) as independent variables, adjusting for medication use (Table 3).

When analyzing the unadjusted results, the odds of children being fussy eaters did not increase for children with ASD, ADHD, or depression versus no disorder. The odds of children being fussy eaters increased by a factor of 5.58 in the crude analysis when having anxiety (95% CI: 1.50–20.77, *p* = 0.010), compared to those who did not have any disorders. When adjusting for medication use, the odds of being a fussy eater lowered to 4.11 (95% CI: 1.02–16.58, *p* = 0.046) when having anxiety.

## 4 DISCUSSION

The purpose of this study was to investigate fussy eating in a clinical sample of Icelandic children with obesity. The primary aim was to investigate the prevalence of fussy eating in this sample. The main results indicated that nearly half of the parents considered their child a fussy eater. Over a quarter of parents considered their child to be food neophobic and considered their child to consume a narrow range of foods. Although prevalence rates of fussy eating are highly varied throughout the literature,^14^, ^35^, ^61^ this was a lower overall rate than expected based on previous reports on fussy eating in children with obesity.^22^ The prevalence rates were also lower than in some studies on children with ASD,^35^ although similar to the 40% found in the twin study of Swedish 9–12‐year olds with ASD and ADHD.^34^ To the authors' best knowledge, no reports exist of fussy eating in a sample of children with obesity and anxiety or depression. The reasons for lower overall prevalence rates are not immediately clear. Possibly, the use of more detailed and varied questions on fussy eating, rather than just asking whether the child is a fussy eater or not, gives parents a broader perspective on what is meant by being a fussy eater.

Over a third of children rejected bitter and sour tastes with only a small proportion of the children rejecting sweet (1.9%) and salty tastes (7.9%). This is in concordance with studies showing children with fussy eating accepting foods that are sweet and rejecting foods with bitter tastes.^18^, ^61^ Some researchers have indicated that genetic predispositions may determine bitter taste sensitivity associated with a stronger preference for sweeter tasting foods and drinks,^62^ perhaps paradoxically due to increased tolerance of sweetness,^63^ and disliking bitter tastes which may be found in vegetables.^64^ The acceptance of sweet and salty tastes and rejecting bitter tastes may also indicate a preference for unhealthier foods which may reflect food choices within our sample of children with obesity.^20^, ^21^, ^27^, ^28^ Although the present study did not ask about types of foods eaten, bitter taste especially characterizes vegetables which is a known hurdle for many children.^16^, ^17^, ^18^, ^35^, ^65^ Furthermore, children with obesity in previous studies have been more likely to consume sweeter foods than children in the healthy weight range,^20^, ^27^ which is perhaps reflected in the small proportion of children in the present study who rejected sweet tastes.

A secondary aim was to investigate whether children with obesity, with or without comorbid disorders, were more likely to be fussy eaters, compared to those who did not have these disorders. The prevalence rates of neurodevelopmental and psychological disorders in this study were similar to recent findings of 5.0–16.5‐year‐old children attending obesity treatment in Sweden.^5^ Comparison of parents' estimate of children's food‐related behaviors based on disorders revealed a significant association for children with ASD and *Narrow range*, *Food neophobia*, and the combination variable *Fussy eating*. Similar results were found for children with anxiety, with significant associations for *Neophobia* and the combination variable *Fussy eating*. Significant associations were also found for children with depression and *Fussy eating*, but no association between any of the food‐related behaviors and ADHD.

Anxiety in disrupted food‐related behaviors has been reported in children with ASD,^44^ and with the comorbid nature of our clinical sample, taking a closer look at anxiety, as presented in children with obesity and fussy eating, may be of importance. In children with fussy eating, anxiety may be elevated with parental pressure and negative comments regarding difficult food behaviors, creating high levels of anxiety which have been shown to increase fussy eating in children.^41^, ^44^ Furthermore, if children's behaviors are difficult during mealtimes,^66^, ^67^, ^68^ this may possibly cause changed dietary behavior, resulting in raised stress levels in parents,^26^, ^69^ which in turn may contribute to and maintain obesity in children.^67^ However, not all authors agree on the association between fussy eating and child weight status being influenced by parental practices.^14^ It may also be easy to overstimulate children who are already susceptible to anxiety and overstimulation.^70^, ^71^ For example, children with ND, especially those with ASD, can be easily overstimulated when pressured to taste previously refused or unfamiliar food, which in turn can generate stress and amplify difficult eating behaviors.^72^, ^73^, ^74^, ^75^ Using a gentle sensory‐based, repeated exposure approach to overcome fussy eating may be particularly well suited for children who are already susceptible to anxiety and overstimulation.^76^, ^77^, ^78^, ^79^, ^80^ It is important to note that nearly a quarter of the children in our study had anxiety or ADHD, of which more than half had both disorders. Similarly, the majority of children with ASD had comorbid ADHD and anxiety. The complex nature of our clinical study sample poses several treatment challenges, which place focus on the importance of investigating new methods for increasing acceptance of healthier foods, especially fruit and vegetables.^10^, ^81^, ^82^, ^83^

Several interventions have been developed with the goal of increasing fruit and vegetable intake in children^10^, ^36^, ^84^, ^85^; however, none of these studies have included children with obesity, anxiety, and fussy eating. Exploring the role of anxiety or fear toward textures and tactile qualities as a possible mechanism in fussy eating might provide insight into the eating behavior challenges facing some children with sensory sensitivities.^80^ Furthermore, even though the odds of fussy eating were not raised for children with ASD, ADHD, or depression, the addition of medication use lowered the odds of fussy eating for children with anxiety. The comorbid nature of children's diagnoses status in obesity treatment must not be overlooked, especially since many of the children with complex disorders may use psychotropic medication, which might affect appetite or even taste perception.^53^, ^54^

The results indicated that the independent variables, ASD, ADHD, and depression, were not associated with increased odds of fussy eating in children with obesity. This may be due to some of the children having multiple disorders, although the variance inflation factor (VIF) was less than 1.7 for all variables entered in the regression model. VIF of lower than 2.5 is generally accepted as a conservative number for detecting multicollinearity.^86^ However, the small size of our study sample may also adversely affect the regression model.^87^

The present study has many strengths. Primarily, the study is the first to investigate the prevalence of fussy eating in a clinical sample of children with obesity and ASD, ADHD, anxiety, or depression. The study is also the first to report on children in obesity treatment and their association with fussy eating and ASD, ADHD, anxiety, or depression. The inclusive nature of the obesity treatment program in Iceland might provide useful insight into the prevalence of fussy eating in a clinically challenging and diverse group of participants, and furthermore, might aid understanding regarding treatment approach, especially in terms of anxiety, sensory issues of taste, and fussy eating.

Whilst the findings of this research add to the body of knowledge regarding a clinically diverse group of children with obesity, this study has several limitations. These include a cross‐sectional, retrospective design and parent‐reported data on dietary behaviors. The main outcome measure was based on the Adult Picky Eating Questionnaire,^60^ which has not been validated for use on children. At that time, no other translated food behavior measures existed in Iceland. The sample size was relatively small and, although not self‐selected, likely biased by a greater proportion of parents who had interest in, or worries regarding, their children's eating behaviors. Although the response rate was 54.7%, this included only those that had provided their contact details for the hospital database between 2011 and 2016. No information was available for those who chose not to participate. The study did not analyze children's medication types, duration, or dosages; however, considering that a quarter of the children had two disorders or more, it is likely that many of the children were on a mixture of psychotropic medications which might affect appetite or preferences toward food.^4^, ^88^, ^89^ The children had clinically diverse comorbid disorders, and all had severe obesity. Therefore, they do not necessarily represent children attending obesity treatment elsewhere, despite representing our patient population well. Parental psychopathology or fussy eating was not investigated, both of which can influence parents' estimate of their children's fussy eating. As with many studies on fussy eating, the terms were broadly defined and did not rely on dietary intake data. However, the questions used in the present study were based on a published eating behavior questionnaire,^60^ and common methods of measuring dietary intake may not be sensitive to certain aspects of fussy eating. A combination of both methods might be optimal for future research. Direct comparisons with prevalence rates of fussy eating in children attending obesity treatment were not possible as the authors of the present study are not aware of any similar reports. Finally, no comparison group was included in terms of children without obesity.

## 5 CONCLUSION

Despite the limitations, this study adds insight into a clinically diverse and inclusive group of children attending obesity treatment. The raised odds of fussy eating in children with severe obesity and anxiety highlight the need for a tailored treatment approach in terms of nutrition behavior change in this vulnerable group of children. Anxiety may be associated with fear toward new foods, food refusals, and behavioral challenges during mealtimes, which may cause complications in acceptance of healthy foods such as fruit and vegetables. To the authors' best knowledge, there are no current treatment options available for children with obesity, fussy eating, and anxiety. Finally, we encourage replication of our research with a larger study sample.

## CONFLICT OF INTEREST

No conflict of interest was declared. The authors alone are responsible for the content and writing of the article.

## AUTHOR CONTRIBUTIONS

Anna S. Olafsdottir and Urdur Njardvik were responsible for the study concept and design of the study. Berglind Brynjolfsdottir, Urdur Njardvik, and Anna S. Olafsdottir acquired the data. Sigrun Thorsteinsdottir, Urdur Njardvik, Ragnar Bjarnason, and Anna S. Olafsdottir analyzed and interpreted the data. Sigrun Thorsteinsdottir performed the statistical analysis and drafted the initial manuscript. The manuscript was revised and reviewed by Sigrun Thorsteinsdottir, Anna S. Olafsdottir, Berglind Brynjolfsdottir, Ragnar Bjarnason, and Urdur Njardvik.
